# Preinvasive Colorectal Lesions of African Americans Display an Immunosuppressive Signature Compared to Caucasian Americans

**DOI:** 10.3389/fonc.2021.659036

**Published:** 2021-04-27

**Authors:** Kristin Wallace, Georges J. Nahhas, Christine Bookhout, David N. Lewin, Chrystal M. Paulos, Nana Nikolaishvili-Feinberg, Stephanie M. Cohen, Silvia Guglietta, Ali Bakhtiari, E. Ramsay Camp, Elizabeth G. Hill, John A. Baron, Jennifer D. Wu, Alexander V. Alekseyenko

**Affiliations:** ^1^ Hollings Cancer Center, Medical University of South Carolina (MUSC), Charleston, SC, United States; ^2^ Department of Public Health Sciences, MUSC, Charleston, SC, United States; ^3^ Department of Psychiatry and Behavioral Sciences, MUSC, Charleston, SC, United States; ^4^ Department of Pathology, University of North Carolina School of Medicine, Chapel Hill, NC, United States; ^5^ Department of Pathology and Laboratory Medicine, MUSC, Charleston, SC, United States; ^6^ Department of Microbiology/Immunology, Emory University School of Medicine, Atlanta, GA, United States; ^7^ Department of Surgery, Emory University School of Medicine, Atlanta, GA, United States; ^8^ Department of Medicine, University of North Carolina School of Medicine, Chapel Hill, NC, United States; ^9^ Feinberg School of Medicine, Northwestern University, Chicago, IL, United States; ^10^ Bioinformatics Center, MUSC, Charleston, SC, United States; ^11^ Department of Oral Health Sciences, MUSC, Charleston, SC, United States; ^12^ Department of Healthcare Leadership and Management, MUSC, Charleston, SC, United States

**Keywords:** race, disparities, colorectal adenomas, immune infiltrate, immune cells

## Abstract

**Background:**

African Americans (AAs) have higher colorectal cancer (CRC) incidence and mortality rate than Caucasian Americans (CAs). Recent studies suggest that immune responses within CRCs contribute to the disparities. If racially distinct immune signatures are present in the early phases of carcinogenesis, they could be used to develop interventions to prevent or slow disease.

**Methods:**

We selected a convenience sample of 95 patients (48 CAs, 47 AAs) with preinvasive colorectal adenomas from the surgical pathology laboratory at the Medical University of South Carolina. Using immunofluorescent-conjugated antibodies on tissue slides from the lesions, we quantified specific immune cell populations: mast cells (CD117^+^), Th17 cells (CD4^+^RORC^+^), and NK cell ligand (MICA/B) and inflammatory cytokines, including *IL-6, IL-17A*, and *IFN-γ*. We compared the mean density counts (MDCs) and density rate ratios (RR) and 95% CI of immune markers between AAs to CAs using negative binomial regression analysis. We adjusted our models for age, sex, clinicopathologic characteristics (histology, location, dysplasia), and batch.

**Results:**

We observed no racial differences in age or sex at the baseline endoscopic exam. AAs compared to CAs had a higher prevalence of proximal adenomas (66% *vs.* 40%) and a lower prevalence of rectal adenomas (11% *vs.* 23%) (p =0.04) but no other differences in pathologic characteristics. In age, sex, and batch adjusted models, AAs *vs.* CAs had lower RRs for cells labeled with IFNγ (RR 0.50 (95% CI 0.32-0.81); p=0.004) and NK cell ligand (RR 0.67 (0.43-1.04); p=0.07). In models adjusted for age, sex, and clinicopathologic variables, AAs had reduced RRs relative to CAs for CD4 (p=0.02), NK cell ligands (p=0.01), Th17 (p=0.005), mast cells (p=0.04) and IFN-γ (p< 0.0001).

**Conclusions:**

Overall, the lower RRs in AAs *vs.* CAs suggests reduced effector response capacity and an immunosuppressive (‘cold’) tumor environment. Our results also highlight the importance of colonic location of adenoma in influencing these differences; the reduced immune responses in AAs relative to CAs may indicate impaired immune surveillance in early carcinogenesis. Future studies are needed to understand the role of risk factors (such as obesity) in influencing differences in immune responses by race.

## Introduction

Colorectal cancer (CRC) is the third most common malignancy among men and women in the US and the second leading cause of cancer death (1). African Americans (AAs) experience higher incidence and mortality from CRC than Caucasian Americans (CAs), especially at younger ages (2–5). Although the disparities are not fully understood, we found that racial differences in the immune landscape of CRC’s play an essential role in patient survival (6). However, whether race-related differences in immune responses are present in the early phases of the carcinogenesis process is unknown (7–9).

The immune system’s role in controlling the growth of established CRC or limiting metastatic expansion is well documented (7, 10, 11). Higher densities of tumor-infiltrating lymphocytes (TILs) with cytotoxic or effector properties (such as Th1, CD8+Tcells, NK cells) are associated with lower recurrence, and better prognosis (10, 12, 13), whereas a greater infiltration with inflammatory Th17 or IL17a cytokines are associated with poorer outcomes (7, 14, 15). Recent evidence has identified lower cytotoxic responses (e.g., Granzyme B, *IFNG*) in CRCs of AAs compared to CAs (16–19), yet higher expression for inflammatory or markers of exhausted T-cells (19). Understanding whether racial differences in immune signatures are evident in earlier phases of carcinogenesis have important consequences for primary and secondary prevention (20, 21).

Earlier data has pointed to the importance of clinicopathologic features (location, histology, grade) in shaping immune responses in colorectal neoplasms (22–24). These features also differ in prevalence by race (4, 5, 25) suggesting the potential for confounding. In the present study, we compared a diverse group of immune cell markers and cytokines in colorectal adenomas from AAs and CAs. We hypothesized that AAs compared to CAs would present at diagnosis with lower density counts for cytotoxic cells/cytokines (NK ligands, mast cells, IFN-γ) and higher inflammatory responses (Th17, Il17a) based on published data in invasive disease (18, 19). Specifically, we compared mean density counts (MDCs) and density rate ratios (RR and 95% CI) for mast cells (CD117), Th17 cells (CD4/RORC), CD4 helper T-cells, NK cell ligands (MICA/B), and cells labeled with inflammatory cytokines, including IL-6, IL-17A, and IFN-γ while adjusting for potential confounding clinicopathologic characteristics.

## Materials and Methods

In this cross-sectional study, we used the Medical University of South Carolina (MUSC) pathology laboratory information system CoPath (Cerner Corporation, Kansas City, MO), to identify a convenient sample of colorectal adenomas excised from patients who underwent a sigmoidoscopy or colonoscopy with polypectomy between October 2012 and May 2016. Patient samples were excluded if the lesion was < 5 mm, as estimated by the study pathologist (SS), or if there was a known familial hereditary syndrome (FAP or Lynch syndrome). The MUSC Institutional Review Board has approved the research study (IRB # PRO-00007139).

### Select of Patient Cohort

The Co-Path system was queried to identify patients diagnosed with an advanced colorectal adenoma. We included search terms colorectal, colon or rectal, adenoma or polyp, as well as high-grade dysplasia, focally high-grade dysplasia, sessile, or traditional serrated adenoma with high-grade dysplasia, or dysplasia). We identified 126 patients (152 lesions) who met the initial screening criteria (dates, advanced histology, colon or rectal polyp, or adenoma) in our search of the Co-Path system. Cases were excluded if the lesion was of non-colonic origin, < 5 mm, as estimated by the MUSC pathologist, or a known familial hereditary syndrome (FAP or Lynch syndrome). Of these, 102 patients were confirmed as having at least one pre-invasive colorectal adenoma with sufficient tissue to be analyzed; 14 of these patients had more than one lesion present at diagnosis. We selected 95 analytic cases (48 CAs, 47 AAs) with at least one conventional adenoma per patient (i.e., tubular, tubulovillous, or villous histology) for the current analysis. We excluded patients (n=7) with a serrated histology lesions (sessile or traditional) index lesion because of potential differences in the prevalence of serrated histology by race (26) and immune infiltrate (27, 28).

For all cases, we abstracted personal characteristics (age at diagnosis, sex, race) from the electronic medical records and clinicopathologic data (anatomic location, grade, degree of dysplasia) from CoPath. To ensure uniformity of diagnoses, an independent pathologist (CB) reviewed all cases using a newly prepared Hematoxylin and Eosin (H&E) slide. The pathologist was blind to any patient or clinical information associated with the lesions and documented the dominant histologic pattern within the adenoma (tubular adenoma, tubulovillous adenoma, villous adenoma) and graded the villous component (0-100%). The pathologist also identified the lesion’s grade according to the most dysplastic area on the slide (i.e., none, low, focally high, high). Each patient contributed one lesion per analysis.

### Immunofluorescence (IF) Optimization, Staining, and Scanning Procedures

The University of North Carolina Translational Pathology Laboratory (TPL) performed the immunofluorescence (IF) multiplex staining. Prior to initiating the IF procedures, all antibodies were optimized using positive (e.g., tonsil) and negative control tissues as recommended by the vendors. A small number of colorectal polyps (similar in size, age and histology to the polyps in the study cohort) and invasive colorectal cancers were also stained and analyzed to demonstrate feasibility. All stains were reviewed by the study immunologist (JW), the pathologist (DL), a cancer epidemiologist (KW), and TPL Director (NNF) to ensure agreement and proper staining of cell types (e.g., visual inspection by a pathologist that CD4 was staining lymphocytes). Consecutive duplex (CD117-MICA and CD4-RORC) or triplex (IFN-γ, IL-6, IL-17A) IF stains were performed in the Leica Bond-Rx fully automated staining platform (Leica Biosystems Inc., Norwell, MA). Slides were dewaxed in Bond™ Dewax solution (#AR9222) and hydrated in Bond Wash solution (#AR9590). The application order of the pretreatment and staining steps, including epitope retrieval (ER), peroxidase and protein blocking, primary and secondary antibodies, and the Tyramide Signal Amplification (TSA), are shown in **Supplemental Table 1**. The ER for the 1st targets (MICA/B, IL-6) was maintained for 20 minutes in Bond ER solution 1 at pH 6.0 (#AR9661) and in ER solution 2 at pH 9.0 (#AR9640) for CD4; and all other targets (2nd and 3rd) for 10 min in ER solution 1. The ER was followed with 10 minutes of endogenous peroxidase blocking using freshly made 3% H_2_0_2_ (Fisher BP2633-500) in methanol (Fisher A433P-4). Stains were completed in succession from 1^st^ to 3^rd^. Slides were counterstained with Hoechst 33258 (# H3569, Life Technologies, Carlsbad, CA) and mounted with ProLong^®^ Gold Antifade Mountant (#P36930, Life Technologies). Positive and negative controls (in which all primary antibodies were omitted) were included in each staining run. Single stain controls (with one primary antibody omitted) were also included to check the cross and detection’s cross-reactivity.

### Automated Image Analysis

We imaged slides in the Leica Aperio-FL (Leica Biosystems) in the Hoechst (blue), Cy2 (green/cyan), Cy3 (green), Cy5 (red) channels. The pathologist and analyst manually annotated regions containing tumor or non-tumor glandular colon tissue on the entire image, only excluding regions containing tissue artifacts or non-cellular areas. Annotated tissue regions were digitally analyzed using Tissue Studio Composer software version 2.7 (Tissue Studio Library version 4.4.2; Definiens Inc., Carlsbad CA). Tissue Studio software was used to identify all nucleated cells within the area on the image (mm^2^). The software determined whether the average staining intensity was above the background threshold determined by negative regions on test slides for each immune protein marker. Tissue Studio software identified cells that expressed or co-expressed the targeted immune markers. We calculated the total number of nucleated cells within the polyp region and the number of these cells positive for each marker. To signify Th17 cells, we used colocalization of CD4 and RORC. Representative stained images and software generated images are shown in **Supplementary Figure 1**.

### Statistical Analysis

For cases, the primary endpoint was the count of immune markers within adenomas. We modeled the log count of positive cells using a negative binomial generalized linear model (GLM), with the logarithm of the sample area included as a model offset for each analyte (29). Briefly, in GLMs for count response variables, the offset is an adjustment term (equivalent to a covariate with unit slope) included in the model whenever the expected count is proportional to an index. Here, we expect the labeled cell counts to be proportional to the spatial area of nucleated cells, a feature that must be accounted for to appropriately isolate the effects of model covariates. Race was included in all models as the primary independent variable of interest, and group comparisons of mean immune marker counts were performed using model-based contrasts (AAs vs. CAs).

Univariable and multivariable models estimated the mean density counts (MDC) and density count rate ratios (RR) and their 95% confidence intervals (CI) for AAs and CAs for each marker type. All multivariable models were adjusted for age (continuous), sex, and IF batch (indicator variables for six batches) (base model). Additional multivariable models were constructed by adding each of the following clinicopathologic variables individually to the base model: lesion anatomic location (proximal colon, distal colon, rectum), percent of villous component (0-100%), and degree of dysplasia (not high, high-grade); fully adjusted models controlled for age, sex, batch, location, histology, and dysplasia. P-values for the differences in models were based on Wald tests. We also tested the interactions between race and clinicopathologic variables and immune counts using the likelihood ratio test. All tests were two-sided, with a significance level (alpha) of p <0.05.

## Results

We analyzed preinvasive lesions from 95 patients (48 CAs, 47 AAs). Univariate associations of personal and clinicopathologic characteristics with race are shown in **Table 1**. AAs had a higher prevalence of proximal adenomas (66% *vs.* 40%) than CAs and a lower prevalence of rectal adenomas (11% vs. 23%) (p =0.04). AAs compared to CAs presented with a similar prevalence of high-grade dysplasia lesions compared to CAs (53% vs. 40%, p=0.18). No difference was detected in the percent of villous histology by race (p=0.96).

**Table 1:** Patient and lesion characteristics at diagnosis in African Americans (AAs) and Caucasian Americans (CAs).

Baseline Variables	AAs	CAs	*p*-value*
	N = 47	N = 48	
Age, mean (SD)	63.2 (9.5)	63.3 (10.1)	0.96
Sex, n (%)			0.58
Female	18 (38)	21 (44)	
Male	29 (62)	27 (56)	
Villousness, n (%)			0.96
0-25%	12 (26)	11 (23)	
26-75%	18 (38)	19 (40)	
76%+	17 (36)	18 (37)	
Location, n (%)			0.04
Proximal	31 (66)	19 (36)	
Distal	11 (23)	17 (40)	
Rectal	5 (11)	11 (23)	
Dysplasia, n (%)			0.18
High	25 (53)	18 (40)	
Not High	24 (47)	34 (60)	

*p-values determined using chi-square tests for categorical variables and t-tests for continuous.

**Table 2** shows the MDCs for each immune marker by race and the RR (95% CI) of the immune markers and race in the multivariable models. In age, sex, and batch adjusted models, AAs had lower RRs for cells labeled with IFNγ (p=0.01) and NK cell ligand (p=0.07) than CAs. In the multivariable models additionally adjusted for colonic location, AAs compared to CAs had significantly lower RRs for cells labeled with CD4, Th17, NK ligands, mast cells, and IFNγ. Further adjustment for percent villous histology and degree of dysplasia did not materially alter the RRs further (i.e., fully adjusted models, **Table 2**). There were no statistically significant interactions between race and any clinicopathologic variables (location, degree of dysplasia, percent villous histology) on the immune counts.

**Table 2:** Mean density counts (MDC) and density rate ratios (RR) in African American (AA) and Caucasian American (CA) tumor immune markers.

Immune markers	AAs N=47 MCDs^1^ (95% CI)	CAs N=48M CDs^1^ (95% CI)	AAs *vs.* CAsRR^2^ (95% CI)	AAs *vs.* CAsRR^3^ (95% CI)
CD4+	81 (59-110)	103 (75-140)	0.78 (0.49-1.26)	**0.53 (0.33-0.87)**
Th17	65 (46-90)	89 (64-125)	0.72 (0.43-1.20)	**0.43 (0.26-0.74)**
NK cell Ligand	1288 (959-1729)	1931 (1443-2583)	0.67 (0.43-1.04)	**0.57 (0.37-0.88)**
IL17a	254 (199-325)	197 (154-251)	1.29 (0.90-1.85)	1.33 (0.93-1.91)
IFNγ	1065 (781-1451)	2112 (1556-2866)	**0.50 (0.32-0.81)**	**0.40 (0.25-0.65)**
IL6	749 (599-938)	898 (720-1121)	0.83 (0.60-1.17)	0.79 (0.58-1.08)
Mast Cells	44 (32-60)	62 (45-83)	0.71 (0.45-1.13)	**0.63 (0.41-0.97)**

^1^Mean density counts (MDC) per mm^2^ adjusted for age, sex, batch. ^2^ RR AA vs. CA (referent) adjusted for age, sex, batch ^3^RR AA vs. CA (referent) adjusted for age, sex, location, degree of dysplasia, percent villous histology, and batch. P-value for difference by race were determined using Wald statistics. P-values < 0.05 are bolded.

## Discussion

Overall, we observed few differences in the prevalence of personal or adenoma characteristics by race at the endoscopic exam. The notable exception being that AAs, compared to CAs, had a higher prevalence of proximal neoplasia. For the immunologic markers, AAs had significantly lower CD4, Th17, mast cells, NK ligands, and *IFN-γ* in the fully adjusted models than CAs. IL17a was non-significantly higher in the same race comparison. Our results point to pervasive differences in immune densities in preinvasive lesions by race.

A few studies (30–34) have described the immune cell contextures within preinvasive lesions, but none have considered these differences by race. Our results point to a dampened immune response in AAs compared to CAs across multiple analytes known to have different functions in the tumor bed. For example, NK ligands, mast cells, and IFNγ play essential roles in orchestrating the cytotoxic (killing) response in the tumors, enabling the host to recognize and eliminate neoplastic cells (7, 14, 35). Reduced cytotoxic responses in AAs *vs.* CAs could suggest compromised immune surveillance capability and the promotion of an environment favorable for tumor growth. Our data pointed to a high density of IFNγ markers and NK cell ligands within the tumor bed relative to other cell types suggesting these immune markers may be very active in early carcinogenesis. Other studies have found that the higher densities of Th1 cytokines and NK cells are inversely related to tumor progression and high grade dysplasia (30, 36). The consequences of lower Th17 densities in adenomas of AAs relative to CAs are less clear due to the functional plasticity of Th17 cells. Th17 cells can promote an inflammatory or immunosuppressive tumor environment depending on the cytokine milieu (37–39) but also play a fundamental role in epithelial barrier homeostasis and control of microbial populations in the gut (40–42). The lower Th17 densities in AAs *vs.* CAs could lead to mucosal barrier compromise and increased microbial translocation, while the trend toward higher IL17a responses may reflect an inflammatory reaction to tumor associated microbes (41, 43). Cui and others (32) found IL17a increased from low to high dysplastic lesions, suggesting a positive correlation with tumor progression in early carcinogenesis; no studies have examined Th17 cells in adenomas. More research is needed to understand the benefits/harms of Th17/IL17a in the tumor environment and their role in tumor progression.

Lower cytotoxic or effector T cell responses in CRCs of AAs *vs.* CAs have been observed in a few previous studies in colorectal cancers (16–19). Basa et al. (18) identified lower protein expression of granzyme B in CRCs of AAs compared to CAs. Granzyme B plays a crucial role in the apoptosis of tumor cells and is secreted by many cells, including NK cells (44), CD8+ T-cells (44), and mast cells (45). Other recent data showed that CRCs in CAs vs. AAs had greater expression of cytotoxic genes *GZMB* and *IFNG* (19). In that study, AAs also exhibited a greater immunosuppressive and exhausted T-cell phenotype than CAs and increased expression of antimicrobial inflammatory cytokines (46). There is also evidence of reduced cytotoxic responses (47) or immune suppression (48) in AAs compared to CAs with prostate cancer and non-cancer contexts (49). Whether there are shared risk factors or genetic characteristics influencing these results is not known; however, the racial differences appear to be influenced by tumor location in the colorectum.

The tumors of the proximal colon (compared to distal or rectal) are more apt to be immunologically active (or ‘hot’) (50, 51). In an earlier study in this population six of the seven markers studied (all except IL-17a) had significantly higher density counts in the proximal colon than distal colon or rectum. The higher immune activity in the proximal colon appears to be shaped by many factors, including the porousness of the mucosal barrier, microbiota, and metabolic activity (52, 53). It’s tempting to speculate that differences in the prevalence of risk factors for proximal colon neoplasia (such as diabetes, obesity) (54–56) –which are more common in AAs– contribute to the diminished immune responses we observed in AAs vs. CAs. Metabolic dysregulation, common in obesity and diabetes, is associated with chronic immune activation and overtime, reduced antitumor effector responses, immune cell death, and tumor progression (57–59). AAs compared to CAs have a higher prevalence of proximal neoplasia (24, 60, 61), consistent with our findings in the present study. Previously, we identified a higher prevalence of metabolic risk factors (i.e., obesity, diabetes, hypertension) in AAs vs. CAs in patients undergoing colonoscopy (62, 63). Future studies will be needed to investigate whether the higher prevalence of metabolic risk factors in AAs compared to CAs contributes to the diminished immune responses observed in the present study when adjusting for colonic location.

Our study has several advantages. It is the first study to compare the immune environment in preinvasive colorectal lesions by race. We adjusted our results for important potential confounders such as age, location, grade, histology. We analyzed immune counts in the entire slide (*vs.* cores), which represent the whole tumor. An independent pathologist with no knowledge of the clinical or personal characteristics of the patients provided blinded diagnoses. We are also aware of several limitations of this study. We had a relatively small number of cases. We did not assess immune counts in different regions of the polyp (e.g., stromal, epithelial), which appear important for CRC outcomes (64). Our study lacks information about the ancestral informative markers to characterize the ancestry in our population.

Our results suggest that detailed immunologic profiling of preinvasive lesions will be an essential next step to understand the contributions of different immune cell subsets in CRC risk and prognosis. We lack a comprehensive inventory of immune signatures by race. Although small, our study demonstrates that AAs have an immunosuppressive phenotype at the initial phases of carcinogenesis. If confirmed in a larger cohort of patients, this signature could be used as a prognostic biomarker to guide interventions when therapeutic options may be more effective in preventing progression and recurrence.

## Data Availability Statement

The raw data supporting the conclusions of this article will be made available by the authors, without undue reservation.

## Ethics Statement

The studies involving human participants were reviewed and approved by The Medical University of South Carolina Institutional Review Board has approved the research study (IRB # PRO-00007139). Written informed consent for participation was not required for this study in accordance with the national legislation and the institutional requirements.

## Author Contributions

KW: study concept and design; acquisition of data; analysis and interpretation of data; drafting of the manuscript; critical revision of the manuscript; administrative, technical, or material support; study supervision. GN: analysis and interpretation of data; drafting of the manuscript; statistical analysis. CB: study concept and design; acquisition of data; analysis and interpretation of data. DL: study concept and design; acquisition of data; analysis and interpretation of data; study supervision. CP: analysis and interpretation of data; critical revision of the manuscript NN-F: acquisition of data; analysis and interpretation of data; critical revision of the manuscript; administrative, technical, or material support; study supervision. SC: analysis and interpretation of data; critical revision of the manuscript; administrative, technical, or material support; study supervision. SG: critical revision of the manuscript. EC: critical revision of the manuscript. AB: analysis and interpretation of data. EH: study concept and design; analysis and interpretation of data; statistical analysis. JW: study concept and design; analysis and interpretation of data; drafting of the manuscript; critical revision of the manuscript; obtained funding. JB: study concept and design; critical revision of the manuscript. AA: study concept and design; analysis and interpretation of data; drafting of the manuscript; critical revision of the manuscript. All authors contributed to the article and approved the submitted version.

## Funding

This study was partly funded by grants from National Library of Medicine (Alekseyenko, R01 LM012517). The Biostatistics Shared Resource, Hollings Cancer Center, Medical University of South Carolina (E. Hill, G. El Nanhas P30 CA138313); South Carolina Clinical & Translational Research (SCTR) Institute NIH Grant Numbers UL1 TR000062 and UL1 TR001450.

## Conflict of Interest

The authors declare that the research was conducted in the absence of any commercial or financial relationships that could be construed as a potential conflict of interest.
