# Supplementary figures and images for: Preinvasive Colorectal Lesions of African Americans Display an Immunosuppressive Signature Compared to Caucasian Americans
Source: Front Oncol. 2021 Apr 27;11:659036. doi: 10.3389/fonc.2021.659036 (PMC8112239; doi:10.3389/fonc.2021.659036)

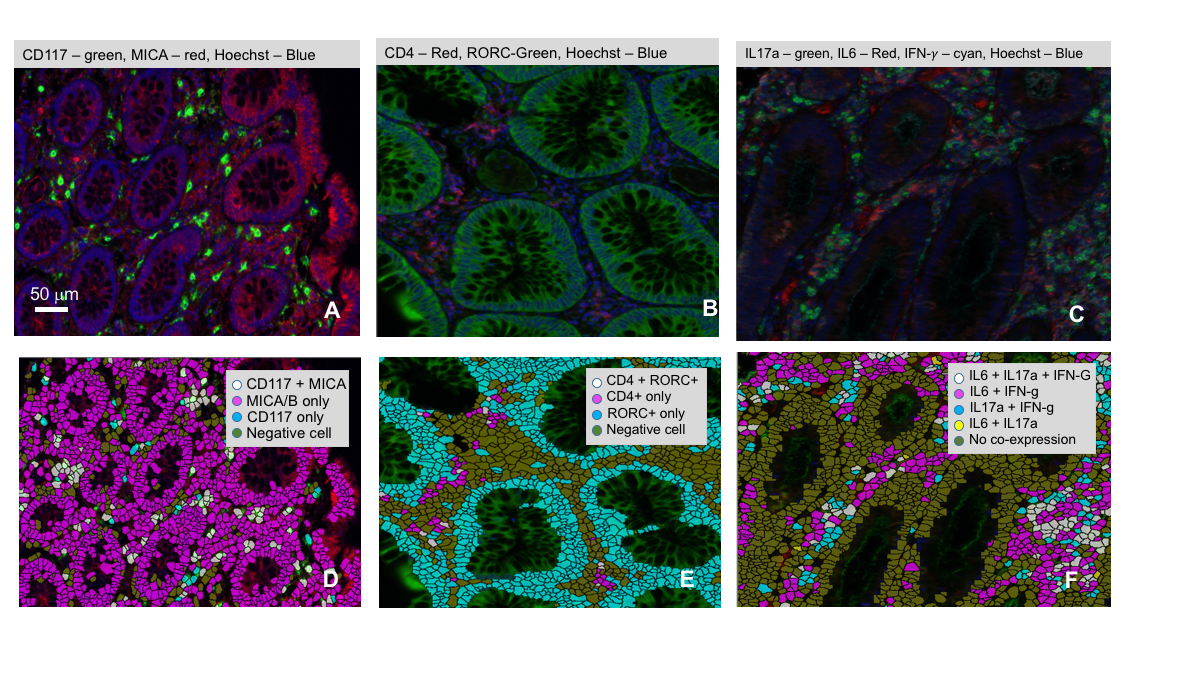

Supplement: Supplementary file 1 [file Image_1.tiff]
