# Supplementary material for: Preinvasive Colorectal Lesions of African Americans Display an Immunosuppressive Signature Compared to Caucasian Americans
Source: Front Oncol. 2021 Apr 27;11:659036. doi: 10.3389/fonc.2021.659036 (PMC8112239; doi:10.3389/fonc.2021.659036)
Supplement: Supplementary file 2 [file Table_1.docx]

**Supplemental Table 1: Mean density counts and RRs in AA vs. CA by age for tumor immune markers.**

|  | Younger age (< 56 years) | |  | Older age (56+ years) | |  |  |
| --- | --- | --- | --- | --- | --- | --- | --- |
| Immune markers | **AAs**  **MCDs (95% CI)** | **CAs**  **MCDs (95% CI)** | **AA vs. CA**  **RR^1^ (95% CI)** | **AAs**  **MCDs (95% CI)** | **CAs**  **MCDs (95% CI)** | **AA vs. CA**  **RR (95% CI)** | **P-for interaction*** |
| CD4+ | 141 (78-255) | 69 (43-111) | 2.05 (0.89-4.70) | 58 (41-84) | 117 (81-169) | 0.50 (0.29-0.88) | 0.03 |
| Th17 | 112 (60-208) | 48 (29-79) | 2.33 (0.97-5.58) | 48 (34-70) | 100 (69-147) | 0.48 (0.27-0.87) | 0.03 |
| NK cell Ligand | 1679 (783-3597) | 1128 (613-276) | 0.96 (0.38-2.48) | 1141 (861-1515) | 1988 (1479-2673) | 0.56 (0.36-0.87) | 0.16 |
| IL17a | 225 (159-320) | 166 (125-221) | 1.36 (0.85-2.18) | 257 (190-348) | 209 (152-287) | 1.23 (0.77-1.97) | 0.57 |
| IFN𝛾 | 1190 (622-2273) | 1052 (627-1766) | 1.13 (0.45-2.81) | 980 (712-1349) | 2211 (1582-3091) | 0.44 (0.27-0.73) | 0.27 |
| IL6 | 756 (410-1379) | 897 (549-1465) | 0.84 (0.36-2.00) | 712 (570-888) | 868 (687-1094) | 0.82 (0.58-1.16) | 0.99 |
| Mast Cells | 36 (20-64) | 53 (33-84) | 0.67 (0.29-1.57) | 40 (28-56) | 66 (46-95) | 0.60 (0.35-1.05) | 0.43 |

^1^Mean density counts (MDC) per mm^2^ and RRs adjusted for sex, batch; *P-value for the interaction between age category (<56, 56+) and race was determined using a likelihood ratio test .
